# The Axonal Guidance Cue Semaphorin 3C Contributes to Alveolar Growth and Repair

**DOI:** 10.1371/journal.pone.0067225

**Published:** 2013-06-20

**Authors:** Arul Vadivel, Rajesh S. Alphonse, Jennifer J. P. Collins, Tim van Haaften, Megan O’Reilly, Farah Eaton, Bernard Thébaud

**Affiliations:** 1 Ottawa Hospital Research Institute, Sprott Center for Stem Cell Research, Regenerative Medicine Program and Children’s Hospital of Eastern Ontario, University of Ottawa, Ottawa, Ontario, Canada; 2 Department of Pediatrics, School of Human Development, Women and Children’s Health Research Institute, Cardiovascular Research Center and Pulmonary Research Group, University of Alberta, Edmonton, Canada; University of Giessen Lung Center, Germany

## Abstract

Lung diseases characterized by alveolar damage such as bronchopulmonary dysplasia (BPD) in premature infants and emphysema lack efficient treatments. Understanding the mechanisms contributing to normal and impaired alveolar growth and repair may identify new therapeutic targets for these lung diseases. Axonal guidance cues are molecules that guide the outgrowth of axons. Amongst these axonal guidance cues, members of the Semaphorin family, in particular Semaphorin 3C (Sema3C), contribute to early lung branching morphogenesis. The role of Sema3C during alveolar growth and repair is unknown. We hypothesized that Sema3C promotes alveolar development and repair. *In vivo* Sema3C knock down using intranasal siRNA during the postnatal stage of alveolar development in rats caused significant air space enlargement reminiscent of BPD. Sema3C knock down was associated with increased TLR3 expression and lung inflammatory cells influx. In a model of O_2_-induced arrested alveolar growth in newborn rats mimicking BPD, air space enlargement was associated with decreased lung Sema3C mRNA expression. *In vitro*, Sema3C treatment preserved alveolar epithelial cell viability in hyperoxia and accelerated alveolar epithelial cell wound healing. Sema3C preserved lung microvascular endothelial cell vascular network formation *in vitro* under hyperoxic conditions. *In vivo*, Sema3C treatment of hyperoxic rats decreased lung neutrophil influx and preserved alveolar and lung vascular growth. Sema3C also preserved lung plexinA2 and Sema3C expression, alveolar epithelial cell proliferation and decreased lung apoptosis. In conclusion, the axonal guidance cue Sema3C promotes normal alveolar growth and may be worthwhile further investigating as a potential therapeutic target for lung repair.

## Introduction

Bronchopulmonary dysplasia (BPD) in premature infants and emphysema represent a major health care challenge because of lack of efficient therapies [1], [2]. A common denominator of these diseases is the absence of injury resolution leading to distorted tissue repair and/or scarring resulting in arrested alveolar growth in BPD and alveolar destruction in emphysema [3].

Understanding how alveoli and the underlying capillaries develop and how these mechanisms are disrupted in disease states are critical for developing effective therapies for lung diseases characterized by alveolar damage [4].

Axonal guidance cues (AGC) are molecules that regulate neural network formation in the nervous system and the outgrowth of axons [5]. During development, specific connections between neurons and between neurons and their non-neuronal targets are determined in part by guidance molecules that allow neuronal growth cones to make selective pathway choices by providing both repulsive and attractive signals. Similar mechanisms may apply to the outgrowth of secondary crests that subdivide the sacculi into alveoli to increase the gas-exchange surface area during alveolar development [6].

Among AGCs, members of the Semaphorin family, in particular class 3 semaphorins (Sema3), contribute to early lung branching morphogenesis. In early lung development, Sema3A is expressed mainly in the distal mesenchyme, Sema3C is expressed mainly in the large airway epithelium, and Sema3F is weakly expressed in both epithelium and mesenchyme [7], [8]. Functional assays suggest that Sema3 contribute to early lung development and have dual effects on branching morphogenesis in fetal lung explants. Exogenous Sema3A inhibits branching, while Sema3C and Sema3F enhance it. Later in gestation and after birth Sema3A is expressed in the terminal epithelium and mesenchyme and Sema3C and Sema3F are expressed in the distal epithelia. These expression profiles suggest that Sema3 signaling may contribute to alveolar development. But their role during this crucial period of lung development remains unexplored.

In addition, little is known about the role of Sema during tissue repair and the few available data derive from literature in the nervous system (reviewed in [9]).

Here we show that: 1) Sema3C contributes to normal postnatal alveolar development because its inhibition leads to arrested alveolar growth; 2) Sema3C signaling is impaired in an experimental model of hyperoxia-induced arrested alveolar growth (mimicking features of BPD) in newborn rats; 3) *in vitro*, Sema3C treatment preserves lung alveolar epithelial cell (AEC) survival in hyperoxia, accelerates AEC wound healing and maintains the ability of lung microvascular endothelial cells to form vascular networks in hyperoxia; 4) *in vivo*, Sema3C treatment preserves alveolar growth in the hyperoxia-induced BPD model in newborn rats.

## Materials and Methods

All procedures were approved by the Institutional Animal Care and Use Committee at the University of Alberta.

### *In vivo* Sema3C Knock down using Small Interfering RNA (siRNA)

Lung Sema3C was inhibited using intranasal Sema3c siRNA (Ambion, Austin, TX) administration (4 µg/g in 2.5 µl/g) [10] to rat pups at postnatal day (P) 4, 7, and 10 [10]–[13].

### Immunoblotting

Protein expression in whole lungs was measured with immunoblotting as previously described [14] using commercially available antibodies.

### Lung Morphometry

Lungs were inflated and fixed *in situ* via the trachea with a zinc formalin solution at a constant pressure of 20 cm H_2_O. After tracheal ligation lungs were removed and placed in fixative overnight, then processed and paraffin embedded. Five µm thick coronal sections were cut and stained with hematoxylin and eosin. Alveolar structures were quantified using the mean linear intercept methods as previously described [14], [15].

### Oxygen-induced Lung Injury

Experimental BPD was induced as previously described [14], [15]. Sprague-Dawley rats (Charles River, Saint Constant, QC, Canada) were exposed to normoxia (21% O_2_) or hyperoxia (95% O_2_, BPD model) from birth to P14 in sealed plexiglass chambers with continuous O_2_ monitoring (BioSpherix, Redfield, NY).

### Quantitative Real-Time Polymerase Chain Reaction (qRT-PCR)

Whole lungs were analyzed by qRT-PCR using specific primers (Applied Biosystems, Foster City, CA) as previously described [14], [15].

### Isolation and O_2_-exposure of Alveolar Epithelial Type 2 Cells (AEC2)

Fetal day 19.5 rat lungs were removed and placed in serum-free DMEM on ice. The trachea was dissected away and the lungs were finely minced. The lung tissue was placed in a digestion solution containing 1 mg/mL Type V collagenase (Sigma, Oakville, ON, Canada) and 20 µg/mL DNase I at 37°C for 30 minutes. The supernatant was removed, centrifuged (5 minutes, 1500 RPM), and the cellular pellet resuspended in DMEM with serum. Fresh digestion solution was added to the remaining lung tissue, and this procedure was repeated until all tissue was digested. Collected cells were filtered through a 50 µm mesh, counted, and set to a concentration of 1.7×10^6^ cells/mL. Cells were seeded on to plastic tissue culture flasks for 45 minutes, after which the supernatant containing the AEC2 was collected and filtered through a 50 µm mesh. This differential plating step was repeated 3 more times to separate fibroblasts from AEC2s [15], [16].

### *In Vitro* AEC2 Cell Viability Assay

After 48 hrs of culture in normoxic (control) or hyperoxic conditions, cell viability was evaluated by measuring the mitochondrial-dependent reduction of colorless 3-(4,5-Dimethylthiazol-2-yl)-2,5- diphenyltetrazolium bromide (MTT) (Invitrogen, Eugene, Oregon, USA) to a colored blue formazan which was dissolved in dimethyl sulfoxide and the absorbance of each sample was spectrophotometrically measured at 550 nm with a Spectra Max 190 (Molecular Devices) microplate reader as previously described [16].

### Wound Healing Assay with Fetal Rat AEC2

Freshly isolated AEC2 were seeded into a plastic 24-well cell culture plate, at a concentration of 10^6^ cells/mL in DMEM (with 20% FBS and 1% PSF). At 36 hours, the cell monolayer was scraped with a p200 pipette tip and medium was replaced with DMEM or DMEM supplemented with Sema3C-FC chimera (R&D Systems) [360 ng/ml]. The surface area of the wound was recorded over time with OpenLab (Quorum Technologies Inc, Guelph, ON, Canada) [15].

### Lung Endothelial Cell Isolation

Pulmonary micro vascular endothelial cells (PMVECs) were isolated from P14 lungs. A single cell suspension - obtained from chopped lung pieces and strained through 70 and 40 mm cell strainers – was washed in DMEM with 10% fetal calf serum, resuspended in phosphate buffered saline (PBS) containing 0.1% (w/v) bovine serum albumin (BSA) and incubated with streptavidin tagged dynabeads (Dynal, Invitrogen, Burlington, ON) pretreated with biotinylated anti-rat CD31 antibody (Abcam, Cambridge, MA). Dynabead tagged CD31 positive cells were selected and snap frozen in liquid nitrogen and stored in −80°C until use.

### Endothelial Network Formation Assay

The formation of cord-like structures by PMVEC was assessed by seeding 80,000 cells/well into 24-well plates coated with Matrigel (BD Biosciences, Mississauga, ON), supplemented with DMEM or DMEM supplemented with Sema3C-FC chimera (R&D Systems, Inc. Minneapolis, MN, USA) [360 ng/ml] and incubated at 37°C for 8 h in normoxia (21%) or hyperoxia (85%). Cord-like structures were observed using an inverted phase contrast microscope (Olympus, Melville, NY) and quantified by measuring the number of intersects and the length of structures in random fields from each well as described [14], [15].

### *In vivo* Sema3C Treatment

Sema3C (R&D Systems,) diluted in sterile distilled water was administered intraperitoneally [17] (25 µg/kg/day) from P4 to P14.

### Immunohistochemistry

Paraffin sections (5 µm, coronal) from the right upper middle lobe (P14 Sema3C knockdown groups) or the left lung (P21 Sema3C treatment groups) were stained for CD68 (all groups) (1∶250, ab125212, Abcam), Myeloperoxidase (MPO) (all groups) (1∶500, ab45977, Abcam), Sema3C (Sema3C treated groups only) (1∶50, NBP1-51281, Novus Biologicals), PlexinA2 (Sema3C treated groups only) (1∶50, sc-25640, Santa Cruz Biotechnology), Myelin Basic Protein (MBP) (Sema3C treated groups only) (M3821, Sigma-Aldrich) and von Willebrand Factor (vWF) (Sema3C treated groups only) (1∶200, A0082, Dako, Glostrup, Denmark). Briefly, sections were deparaffinized in an ethanol series. Antigen retrieval was performed by incubating slides in heated citrate buffer (10 mM pH 6.0) for 30 minutes, followed by blocking of endogenous peroxidase activity in 3% H_2_O_2_/PBS for 20 minutes. Non-specific binding was blocked by 30 minutes incubation with 10% Normal Goat Serum in 0.1% BSA/PBS at room temperature. Primary antibodies were diluted in 0.1% BSA/PBS and incubated with the sections overnight at 4°C. Subsequently, sections were rinsed in PBS and incubated with a biotinylated polyclonal goat-anti-rabbit IgG secondary antibody (1∶200 in 0.1% BSA/PBS, E0432, Dako) for 1 hour at room temperature. The antibody signal was amplified by incubation with Vectastain Elite ABC reagent (PK-7100, Vector Laboratories, Burlingame, CA, USA) for 30 minutes and visualized with 3,3′-Diaminobenzidine (DAB) staining. Tissue sections were counterstained with Mayer’s hematoxylin, dehydrated in an ethanol series and coverslipped. For vWF staining, non-specific binding was blocked with Tris-HCl/NaCl/BSA buffer and vWF antibody was diluted in Dako antibody diluent (S0809, Dako). After overnight incubation with vWF antibody, sections were incubated with biotinylated goat-anti-rabbit IgG secondary antibody (1∶1000, B2770, Invitrogen) diluted in Dako antibody diluent for 1 hour at room temperature. Antibody signal was amplified by incubation with streptavidin-HRP complex (1∶1000 in Dako diluent; S911 Invitrogen) for 30 minutes at room temperature and visualized with DAB staining. Tissue sections were counterstained Mayer’s hematoxylin, dehydrated in an ethanol series and coverslipped.

Sections were analyzed by light microscopy using a Zeiss (Imager.M2) microscope with Axiovision 4 software. All photomicrographs were taken at 200x magnification. Ki67 staining was semi-quantitatively scored by a blinded observer (AV) in three representative high-power fields per animal using the following system: 0 - no staining; 1 - light staining; 2 - medium staining; 3 - strong staining. CD68 and MPO positive cells were counted by a blinded observer (AV) using Image J software (Rasband, W.S., Image J US National Institutes of Health, Bethesda, Maryland, USA) in three representative high-power fields per animal and were subsequently averaged. vWF-positive vessels were counted by a blinded observer (AV) using Image J software in five representative high-power fields per animal and were subsequently averaged.

### Statistics

Values are expressed as the mean±SEM. Statistical comparisons were made with the use of ANOVA. Post hoc analysis used a Fisher’s probable least significant difference test (Statview 5.1, Abacus Concepts, Berkeley, CA). A value of *P*<0.05 was considered statistically significant.

## Results

### Sema3C Inhibition Disrupts Normal Alveolar Development

**Figure 1:**
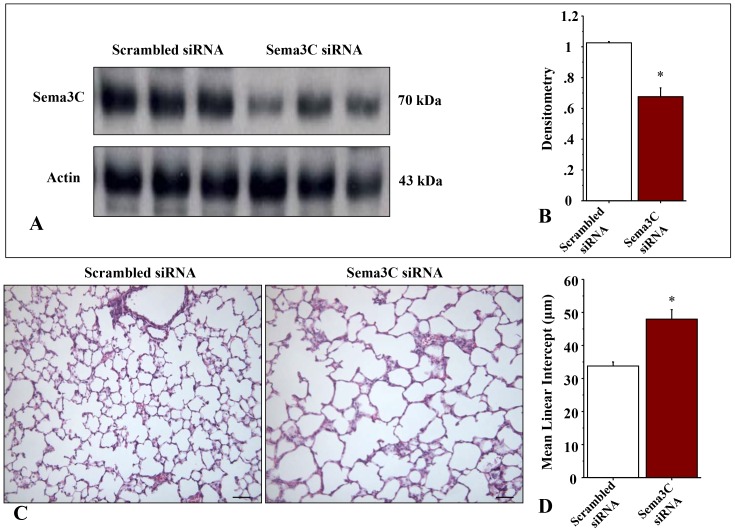
Intranasal Sema3C siRNA disrupts normal alveolar development. A–B. Immunoblotting showing decreased whole lung Sema3C protein expression with intranasal Sema3C siRNA administration as compared to scrambled siRNA at P14 (n = 3 lungs/group, *P<0.05). C. Representative H&E-stained lung sections depicting alveolar simplification (fewer and larger alveoli with decreased secondary septa) reminiscent of BPD in Sema3C siRNA treated rat pups. D. Quantification of the mean linear intercept confirms arrested alveolar growth in Sema3C siRNA treated rat pups compared to scrambled siRNA (control) treated animals (n = 6 lungs/group, *P<0.05, Scale bar 130 µm).

Intranasal administration of Sema3C siRNA efficiently silenced total lung Sema3C protein expression in the neonatal rat lung compared to scrambled siRNA (Figure 1A, B).

Sema3C gene silencing during the critical period of alveolar development resulted in fewer and enlarged air spaces reminiscent of BPD (Figure 1C). Decreased alveolarization was confirmed by the mean linear intercept (Figure 1D). Scrambled siRNA had no effect on lung architecture.

### Sema3C Inhibition Causes Lung Influx of CD68- and MPO-positive Cells

**Figure 2:**
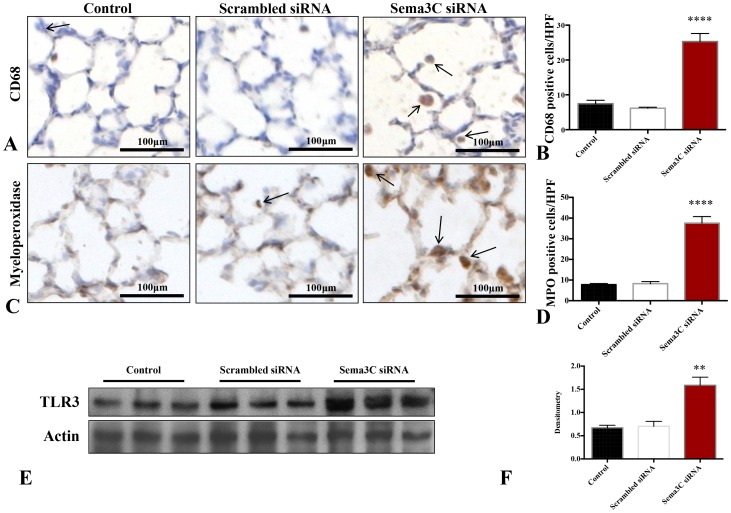
Silencing of Sema3C causes influx of CD68- and myeloperoxidase-positive cells. A. Representative photomicrographs showing immunostaining for CD68 (brown) in the lungs of untreated P14 rats compared to rats treated with intranasal scrambled siRNA and Sema3C siRNA. Arrows highlight CD68-positive cells. Scale bars represent 100 µm. B. The number of CD68-positive cells per high-power field (HPF) increased 3-fold in Sema3C siRNA exposed lungs (n = 3 lungs/group, ****P<0.0001). C. Representative photomicrographs showing immunostaining for myeloperoxidase (MPO) (brown) in the lungs of untreated P14 rats compared to rats treated with intranasal scrambled siRNA and Sema3C siRNA. Arrows highlight MPO-positive cells; scale bars represent 100 µm. D. The number of MPO-positive cells per HPF increased 4-fold in Sema3C siRNA exposed lungs (n = 3 lungs/group, ****P<0.0001). E–F. Immunoblotting of TLR3 in whole lung protein shows increased TLR3 expression in Sema3C siRNA injected rats compared to control and scrambled siRNA exposed rats. (n = 3 lungs/group, **P<0.01).

Sema3C siRNA led to a 3-fold increase of CD68 positive cells, a macrophage marker, when compared to control or scrambled siRNA exposed lungs (Figure 2A–B). Similarly, there was a 4-fold increase in cells expressing neutrophil/monocyte myeloperoxidase (Figure 2C–D). This influx of inflammatory cells was associated with TLR3 up-regulation as compared with untreated and scrambled siRNA treated pups (Figure 2E–F).

### Sema3C Expression is Developmentally Regulated during Normal Lung Growth and Impaired during O_2_-induced Arrested Alveolar Growth

**Figure 3:**
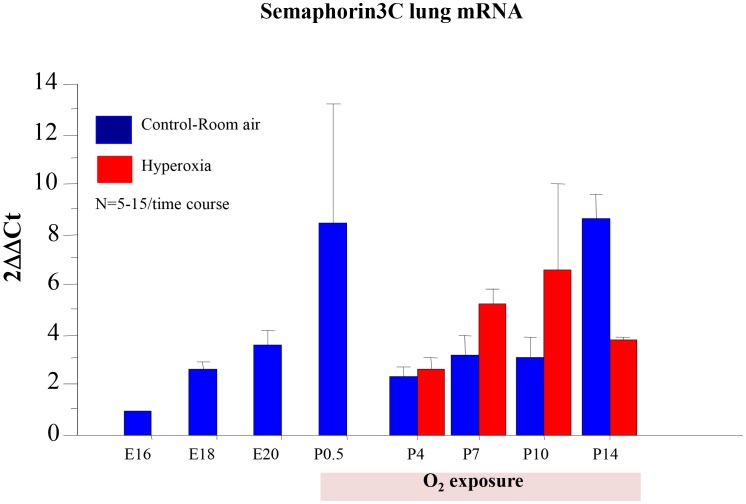
Sema3C signaling is developmentally regulated during lung growth and impaired in O_2_-induced arrested lung growth. Lungs were collected at various time points during embryonic and postnatal lung development and mRNA expression of Sema3C was quantified using qRT-PCR. Sema3C mRNA expression peaked during alveolar development. Sema3C expression was transiently decreased from P10–14 in O_2_-induced experimental BPD (n = 5–15 lungs/time point).

Sema3C mRNA expression peaks during the canalicular stage of lung development and again during alveolar development (Figure 3). Sema3C expression is decreased at P14 towards the end of alveolar development in O_2_-induced experimental BPD.

### Sema3C Protects AEC2 from O_2_-induced Toxicity and Accelerates Wound Healing

**Figure 4:**
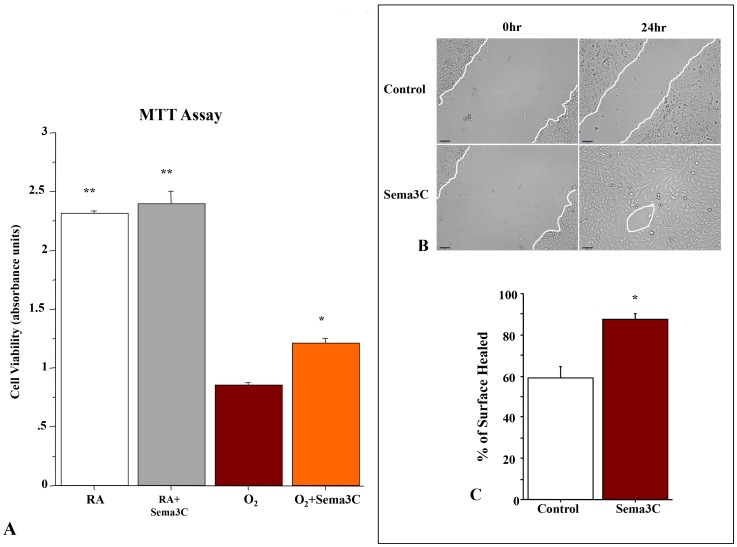
Sema3C preserves type 2 alveolar epithelial cells (AEC2) viability in hyperoxia and promotes wound healing *in vitro*. A. AEC2 were cultured for 48 hrs in room air (normoxic control) or 95% hyperoxia. Mean data of cell viability as assessed by measuring the mitochondrial-dependent reduction of colorless 3-(4,5-Dimethylthiazol-2-yl)-2,5-diphenyltetrazolium bromide (MTT) shows that hyperoxia significantly decreases AEC2 viability as compared with room air exposed cells. Sema3C treatment significantly improves AEC2 viability in hyperoxia (n = 5, **P<0.001 vs O_2_, *P<0.05 vs O_2_). B. Confluent monolayers of AEC2 were damaged using a pipette tip, washed to remove damaged cells, and treated with vehicle or Sema3C. C. Quantification of percent surface area closed confirmed that Sema3C accelerated AEC2 wound closure as compared with vehicle (n = 6/group, *P<0.05, Scale bar 65 µm).

*In vitro*, the viability of freshly isolated AEC2, as assessed by the colorimetric MTT assay, was significantly decreased after 48 hours in 95%O_2_, as compared to room air cultured cells (Figure 4A). Recombinant Sema3C peptide significantly enhanced AEC2 survival in hyperoxia. In addition, Sema3C treatment accelerated AEC2 wound healing in an *in vitro* wound healing scratch assay by 30% compared to vehicle treated AEC2s (Figure 4B–C).

### Sema3C Preserves PMVEC Vascular Network Formation in Hyperoxia *in vitro*

**Figure 5:**
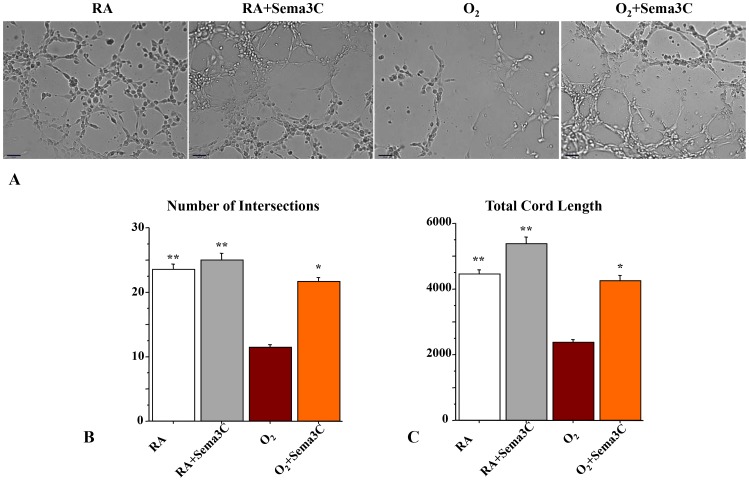
Sema3C promotes pulmonary microvascular endothelial cells (PMVECs) network formation in hyperoxia. A. PMVECs were cultured on matrigel in room air and in hyperoxia. PMVECs form cord-like structures within hours. Hyperoxia significantly decreases the aptitude of PMVECs to form vascular networks. B. C. Quantitative assessment of cord-like structure formation shows a significant decrease in the number of intersects and the total length of cordlike structures in hyperoxia. Sema3C preserved the number of intersects and total cord-structure length. (N = 3/group, **P<0.0001 vs O_2_, *P<0.05 vs O_2_, Scale bar 65 µm).

Freshly isolated PMVECs were exposed to room air or 95% O_2_ in serum-free Matrigel and assessed for the formation of vessel-like networks (Figure 5A). Hyperoxia significantly decreased endothelial cord-like structure formation, whereas Sema3C significantly counteracted the effect of O_2_ and promoted endothelial network formation (Figure 5B).

### Sema3C Treatment Preserves Alveolar and Lung Vascular Growth in O_2_-induced BPD

**Figure 6:**
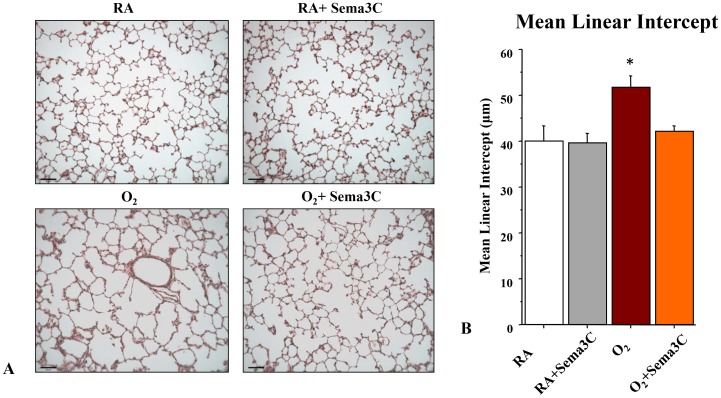
*In vivo* Sema3C treatment prevents arrested alveolar growth in experimental O_2_-induced BPD. A. Representative H&E–stained (A, Scale bar 130 µm) lung sections at P21 showing larger and fewer alveoli in hyperoxia-exposed lungs as compared to lungs of normoxic housed rat pups. Treatment of hyperoxia-exposed animals with Sema3C preserved alveolar structure. B. The mean linear intercept confirms arrested alveolar growth in untreated O_2_-exposed animals and preserved alveolar structure with Sema3C treatment (n = 5/group, *P<0.05 Hyperoxia vs. other groups).

To test the therapeutic potential of Sema3C *in vivo*, neonatal rats exposed to hyperoxia for 14 days were treated with daily intraperitoneal injections of Sema3C. Hyperoxia induced a histological pattern reminiscent of human BPD, characterized by airspace enlargement with simplified and fewer alveolar structures as shown on representative H&E stained section (Figure 6A). Treatment with Sema3C from P4–P14 preserved alveolar development as quantified by the mean linear intercept (Figure 6B). Sema3C treatment had no adverse effects on lung architecture in control animals.

**Figure 7:**
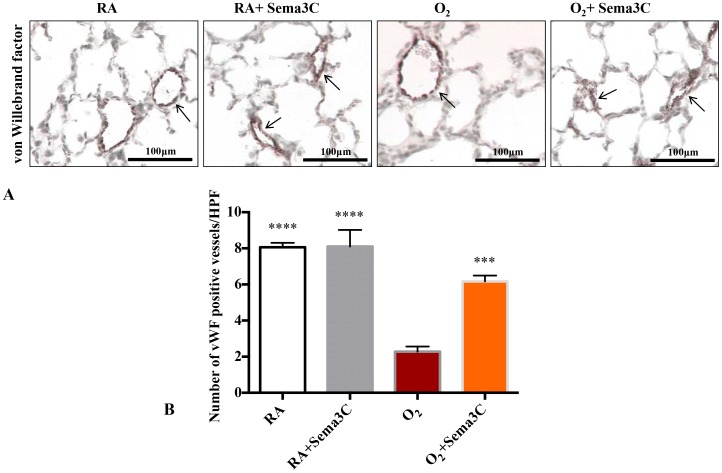
*In vivo* Sema3C treatment prevents O_2_-induced arrested vascular growth. A. Representative photomicrographs showing von Willebrand (vWF) factor staining (brown) in RA (room air), RA+Sema3C, hyperoxia (O_2_) and O_2_+Sema3C exposed lungs. Arrows highlight vWF-positive vessels; scale bars represent 100 µm. B. Mean data quantifying the number of vWF positive vessels between groups. The decrease in the number of vessels per high-power field (HPF) after hyperoxia exposure was prevented by Sema3C treatment (n = 5–7/group, ****P<0.0001 Hyperoxia vs normoxia groups, ***P<0.001 Hyperoxia vs O_2_+Sema3C).

Hyperoxia also lead to an arrest in lung vascular growth as assessed by a decrease in vWF positive lung vessels (Figure 7A–B). Sema3C treatment attenuated the loss of vWF positive cells (Figure 7A–B).

### Sema3C Attenuates Hyperoxia-induced Lung Influx of Inflammatory Cells

**Figure 8:**
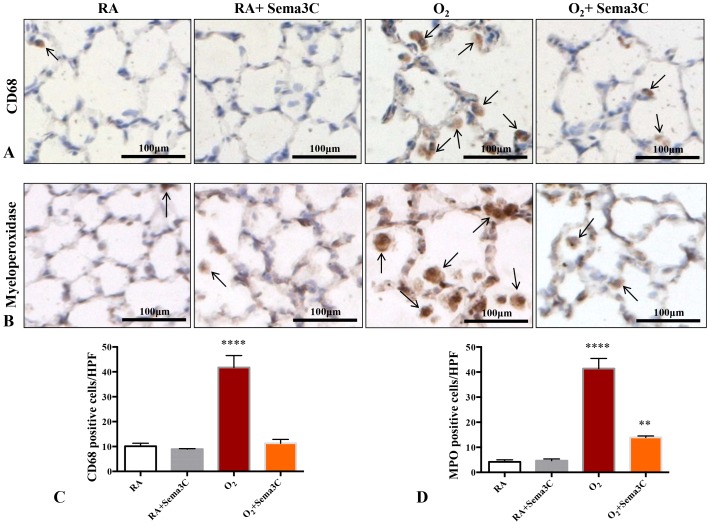
*In vivo* Sema3C treatment prevents O_2_-induced influx of CD68- and myeloperoxidase expressing cells. A. Representative photomicrographs showing CD68 staining (brown) in RA (room air), RA+Sema3C, hyperoxia (O_2_) and O_2_+Sema3C exposed lungs. Arrows highlight CD68-positive cells; scale bars represent 100 µm. B. Representative photomicrographs showing myeloperoxidase (MPO) staining (brown) in RA (room air), RA+Sema3C, hyperoxia (O_2_) and O_2_+Sema3C exposed lungs. Arrows highlight MPO-positive cells; scale bars represent 100 µm. C. Sema3C treatment prevented the hyperoxia-induced increase in CD68-positive cells per high power field (HPF) (n = 5–7/group, ****P<0.0001 Hyperoxia vs other groups). D. Sema3C significantly decreased the hyperoxia-induced influx of MPO-positive cells, although MPO-positive cells/HPF were still increased compared to normoxic control lungs (n = 5–7/group, ****P<0.0001 Hyperoxia vs other groups, **P<0.01 O_2_+Sema3C vs normoxia groups).

Hyperoxia exposure elicited an increase in CD68-positive cells (Figure 8 A–B) and MPO-positive cells (Figure 8C–D). Sema3C treatment significantly attenuated the number of CD68- and MPO-positive cells in the lungs of hyperoxic-exposed animals.

### Sema3C Attenuates Lung Apoptosis and Preserves Alveolar Epithelial Cell Proliferation

**Figure 9:**
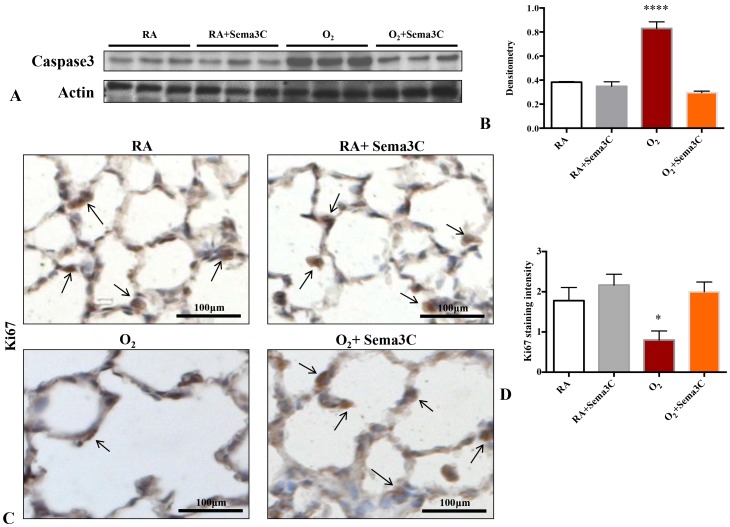
*In vivo* Sema3C treatment prevents O_2_-induced apoptosis and restores proliferation. A–B. Immunoblotting showed increased whole lung caspase 3 expression in hyperoxia exposed lungs, which was prevented by Sema3C treatment (n = 3/group, ****P<0.0001 hyperoxia vs. other groups). C. Representative photomicrographs showing Ki67 staining (brown) in RA (room air), RA+Sema3C, hyperoxia (O_2_) and O_2_+Sema3C exposed lungs. Arrows highlight Ki67-positive cells; scale bars represent 100 µm. D. Ki67 staining intensity was significantly decreased compared to RA-exposed lungs, but was preserved by Sema3C treatment (n = 5–7/group, *P<0.05 Hyperoxia vs other groups).

Hyperoxia exposure resulted in a 2-fold increase in caspase 3 protein levels compared to age-matched normoxia exposed controls (Figure 9A–B). Sema3C treatment significantly attenuated lung caspase 3 expression in hyperoxia. Sema3C had no effect on caspase 3 expression in room air housed rats. Hyperoxia significantly decreased Ki67 expression in alveolar epithelial cells compared to normoxic lungs (Figure 9C–D). Treatment with Sema3C preserved Ki67 expression in hyperoxia. Sema3C had no effect on Ki67 expression in control animals.

### Sema3C Preserved Sema3C and PlexinA2 Expression

**Figure 10:**
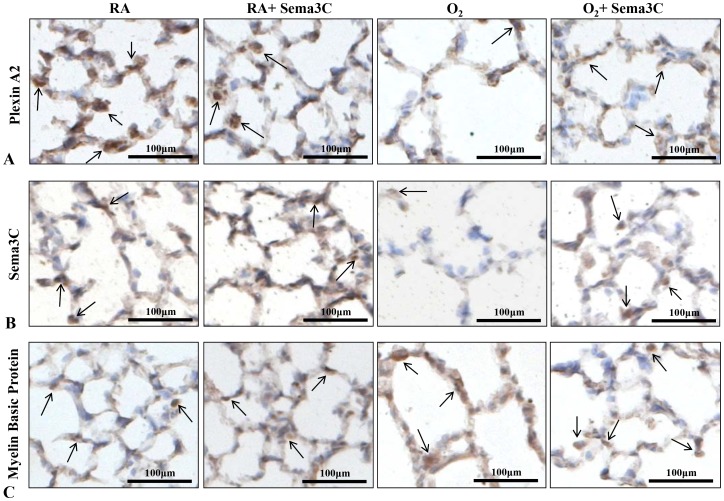
Presence of PlexinA2, Sema3C and myelinated nerves in distal alveoli. A. Representative photomicrographs showing PlexinA2 staining (brown) in RA (room air), RA+Sema3C, hyperoxia (O_2_) and O_2_+Sema3C exposed lungs. Arrows highlight PlexinA2-positive cells; scale bars represent 100 µm. B. Representative photomicrographs showing Sema3C staining (brown) in RA (room air), RA+Sema3C, hyperoxia (O_2_) and O_2_+Sema3C exposed lungs. Arrows highlight Sema3C-positive cells; scale bars represent 100 µm. C. Representative photomicrographs showing myelin basic protein (MBP) staining (brown), a marker for myelinated nerves, in RA (room air), RA+Sema3C, hyperoxia (O_2_) and O_2_+Sema3C exposed lungs. Arrows highlight MBP-positive nerves; scalebars represent 100 µm.

To further confirm that Sema3C had a direct impact on alveolar growth, we stained lung sections for Sema3C and for PlexinA2, a co-receptor specific for Sema3A and Sema3C [18]. Both PlexinA2 and Sema3C expression was significantly decreased in alveolar epithelial cells in hyperoxic rats (Figure 10A–B). Sema3C preserved PlexinA2 expression in hyperoxia (Figure 10A). More intense Sema3C staining was seen in hyperoxic rats treated with Sema3C (Figure 10B). The density of myelinated nerves, as visualized by staining for myelin basic protein, was similar between groups (Figure 10C).

## Discussion

We demonstrate that Sema3C is critical for normal alveolar growth and repair: *in vivo* Sema3C inhibition during the period of alveolar development arrests alveolar growth resulting in histological changes reminiscent of BPD. In experimental O_2_-induced BPD, arrested alveolar growth is associated with disrupted Sema3C signaling. Conversely, treatment with recombinant Sema3C protects AEC2 and PMVEC from O_2_ toxicity and accelerates AEC2 wound healing *in vitro*. *In vivo*, Sema3C administration preserves alveolar growth. This observation suggests a lung protective effect of Sema3C.

### The Axonal Guidance cue Sema3C Contributes to Normal Alveolar Development

In contrast to the large amount of information regarding the genetic control of the dichotomous division of the *conducting airways* in mammals, much less is known about the mechanisms that regulate *alveolar* development. During early branching morphogenesis, the conducting airways grow into the surrounding mesenchyme and many of the regulatory signaling molecules have been identified [19]. Conversely, alveolar septa evaginate inwards into the air space and the genetic control of alveolarization remains poorly understood [20]. One interesting theory is that a repulsive signal pushes the septa inward [6]. During development, specific connections between neurons and between neurons and their non-neuronal targets are determined in part by guidance molecules that allow neuronal growth cones to make selective pathway choices by providing both repulsive and attractive signals [5]. Adhesion receptors transduce the signals from the extracellular substrate to the cytoskeleton, thereby providing the traction necessary to move the growth cone, while guidance cues supply the directional information through attraction or repulsion [5]. Similar mechanisms may apply to the outgrowth of secondary septa during the formation of alveoli [6]. Thus AGCs are appealing candidates in guiding the growth of alveolar septa in the lung during alveolar development.

Sema3C knock out mice die within hours after birth because of cardiovascular defects [21]. In order to overcome early mortality to study the role of this AGC during postnatal alveolar development, we administered intranasal siRNA to neonatal rats to specifically silence lung Sema3C during the critical period of postnatal alveolar development. This strategy effectively silenced lung Sema3C and resulted in arrested alveolar growth. Our findings support the importance of class 3 Sema during postnatal lung development. Becker et al found that Sema3A^−/−^ have increased perinatal mortality [22]. Interestingly, in lungs from the rare Sema3A^−/−^ mice that survived the immediate perinatal period, alveolarization was markedly attenuated. In another study, loss of Sema3-Nrp1 signaling resulted in acute respiratory distress and high neonatal mortality [23]. The lungs of mutants were immature and displayed atelectasis, thickened alveolar septa, reduced capillary density, hypertensive changes in arteriolar walls, anomalous and misaligned pulmonary veins, and reduced pulmonary surfactant secretion. Together, these data provide evidence that class 3 Sema contribute to postnatal lung growth. Sema3C siRNA, but not scrambled siRNA, was associated with increased TLR3 expression and lung influx of inflammatory cells. Thus we cannot exclude that inflammation may also have contributed to the lung phenotype.

We provide additional evidence for the role of Sema3C during alveolar development by showing that Sema3C mRNA expression peaks during the late alveolar stage of lung development (P14). Conversely, Sema3C signaling is disrupted in O_2_-induced arrested alveolar growth: Sema3C mRNA expression is transiently decreased at P14 in hyperoxic-exposed rats compared to rat pups housed in room air. The role of class 3 Sema has also been explored in other lung disease models associated with distal airspace enlargement. Mice with lung-specific deletion of epithelial Nrp1 are more susceptible to cigarette smoke-induced lung injury [24]. Genetic deletion of epithelial Nrp1 in either postnatal or adult lungs resulted in a small increase in airspace size, but when challenged with cigarette smoke, both airspace enlargement and apoptosis of type I and type II alveolar epithelial cells were significantly enhanced in conditionally Nrp1-deficient adult mice. Altogether, these observations suggest that class 3 Sema promote normal alveolar development and are important for maintenance of alveolar homeostasis.

### The axonal Guidance cue Sema3C Prevents Alveolar Damage

The findings above formed the rationale for testing the therapeutic potential of Sema3C to preserve normal alveolar development in a model of O_2_-induced arrested alveolar growth. The repair capabilities of Semaphorins remain relatively unexplored. Most data derive from the central nervous system. Expression studies of Sema3A after lesions of the olfactory nerve or bulbectomy suggest that Sema3A guides regenerating axons, but also contribute to the growth inhibitory characteristics of the scar region after lesions of axon tracts in the central nervous system [25], [26]. Using gain and loss of function experiments in an adult murine demyelination model, Piaton et al found that Sema3A impaired oligodendrocyte precursor cell recruitment to the demyelinated area [27]. In contrast, Sema3F overexpression accelerated oligodendrocyte precursor cell recruitment, and remyelination rate [27]. In a central corneal epithelial debridement model, the expression of Sema3A was markedly increased in basal cells of the newly healed corneal epithelium, and this up-regulation of Sema3A was not associated with cell proliferation suggesting that Sema3A might play a role in the regulation of corneal epithelial wound healing [28].

Here we demonstrate for the first time, both *in vitro and in vivo*, that Sema3C treatment displays lung protective properties. *In vitro*, Sema3C preserved AEC2 viability in hyperoxia and accelerated AEC2 wound closure, suggesting that Sema3C promotes wound healing. Because angiogenesis contributes to alveolar growth [14], we also demonstrate that Sema3C maintains PMVEC network formation in hyperoxia. This is consistent with observations that Sema3C enhances β_1_ integrin function in endothelial cells by inducing its phosphorylation, VEGF_120_ secretion and reduced endothelial cell apoptosis [29].

To explore the therapeutic potential of Sema3C *in vivo*, we used the hyperoxic BPD model of lung injury, hypothesizing that Sema3C would preserve alveolarization. Consistent with our *in vitro* data, Sema3C preserved alveolar and vessel growth in hyperoxic-exposed pups, suggesting Sema3C as promising therapeutic target to prevent alveolar damage. This protective effect was associated with decreased lung neutrophil and macrophages accumulation, which significantly contribute to lung injury in BPD [30], [31]. We found that Sema3C treatment decreased both CD68 and MPO positive cells in lung sections compared to untreated hyperoxia rats. This observation is consistent with the growing evidence of “immune semaphorins” capable of modulating the inflammatory response [32].

Our data indicate that *in vivo* sema3C stimulates proliferation and decreases apoptosis in our oxygen induced lung injury. These findings are consistent with previous studies indicating that Sema3C contributes to endothelial cell guidance, vascular morphogenesis and reduced apoptosis [29]. Also the typical histologic pattern of BPD is a combination of alveolar simplification and decreased pulmonary vessel density [33]. Our immunohistochemistry results using vWF showed that Sema3c treatment restores vessel density in hyperoxia rats. Ki67 is a nuclear protein that is expressed in all active phases (G1, S, G2, and M) of the cell cycle. Ki67 expression is required for cell proliferation and its expression correlates with the mitotic index [34]. Unlike proliferating cell nuclear antigen, Ki67 has a short half-life, which is an important consideration in developing lungs with a relatively high basal rate of cell proliferation. In our current study Sema3C treated lungs showed increased Ki67 expression compared to untreated hyperoxic animals. These findings suggest that the lower expression of Ki67 in hyperoxia might contribute to the arrest in lung growth.

In conclusion, we confirm that the AGC Sema3C is necessary for normal alveolar development. In addition, we show that Sema3C exerts therapeutic benefit in protecting the lung from O_2_-induced alveolar injury. Our findings suggest Sema3C as a molecule worthy of further evaluation as a putative target for lung repair.

## References

[1] Lawn JE, Gravett MG, Nunes TM, Rubens CE, Stanton C (2010) Global report on preterm birth and stillbirth (1 of 7): definitions, description of the burden and opportunities to improve data. BMC Pregnancy Childbirth 10 Suppl 1S1. 20233382 10.1186/1471-2393-10-S1-S1 PMC2841772

[2] Stockley RA, Mannino D, Barnes PJ (2009) Burden and pathogenesis of chronic obstructive pulmonary disease. Proc Am Thorac Soc 6: 524–526. 19741261 10.1513/pats.200904-016DS

[3] Bourbon JR, Boucherat O, Boczkowski J, Crestani B, Delacourt C (2009) Bronchopulmonary dysplasia and emphysema: in search of common therapeutic targets. Trends Mol Med 15: 169–179. 19303361 10.1016/j.molmed.2009.02.003

[4] Beers MF, Morrisey EE (2011) The three R’s of lung health and disease: repair, remodeling, and regeneration. J Clin Invest 121: 2065–2073. 21633173 10.1172/JCI45961 PMC3104764

[5] Barallobre MJ, Pascual M, Del Rio JA, Soriano E (2005) The Netrin family of guidance factors: emphasis on Netrin-1 signalling. Brain Res Brain Res Rev 49: 22–47. 15960985 10.1016/j.brainresrev.2004.11.003

[6] Prodhan P, Kinane TB (2002) Developmental paradigms in terminal lung development. Bioessays 24: 1052–1059. 12386936 10.1002/bies.10177

[7] Ito T, Kagoshima M, Sasaki Y, Li C, Udaka N, et al (2000) Repulsive axon guidance molecule Sema3A inhibits branching morphogenesis of fetal mouse lung. Mech Dev 97: 35–45. 11025205 10.1016/s0925-4773(00)00401-9

[8] Kagoshima M, Ito T (2001) Diverse gene expression and function of semaphorins in developing lung: positive and negative regulatory roles of semaphorins in lung branching morphogenesis. Genes Cells 6: 559–571. 11442635 10.1046/j.1365-2443.2001.00441.x

[9] Koeberle PD, Bahr M (2004) Growth and guidance cues for regenerating axons: where have they gone? J Neurobiol 59: 162–180. 15007834 10.1002/neu.10345

[10] Bitko V, Musiyenko A, Shulyayeva O, Barik S (2005) Inhibition of respiratory viruses by nasally administered siRNA. Nat Med 11: 50–55. 15619632 10.1038/nm1164

[11] Massaro D, Massaro GD, Clerch LB (2004) Noninvasive delivery of small inhibitory RNA and other reagents to pulmonary alveoli in mice. Am J Physiol Lung Cell Mol Physiol 287: L1066–1070. 15234906 10.1152/ajplung.00067.2004

[12] Vadivel A, Abozaid S, van Haaften T, Sawicka M, Eaton F, et al (2010) Adrenomedullin promotes lung angiogenesis, alveolar development, and repair. Am J Respir Cell Mol Biol 43: 152–160. 19738161 10.1165/rcmb.2009-0004OC

[13] Vadivel A, van Haaften T, Alphonse RS, Rey-Parra GJ, Ionescu L, et al (2012) Critical role of the axonal guidance cue EphrinB2 in lung growth, angiogenesis, and repair. Am J Respir Crit Care Med 185: 564–574. 22161159 10.1164/rccm.201103-0545OC

[14] Thebaud B, Ladha F, Michelakis ED, Sawicka M, Thurston G, et al (2005) Vascular endothelial growth factor gene therapy increases survival, promotes lung angiogenesis, and prevents alveolar damage in hyperoxia-induced lung injury: evidence that angiogenesis participates in alveolarization. Circulation 112: 2477–2486. 16230500 10.1161/CIRCULATIONAHA.105.541524

[15] van Haaften T, Byrne R, Bonnet S, Rochefort GY, Akabutu J, et al (2009) Airway Delivery of Mesenchymal Stem Cells Prevents Arrested Alveolar Growth in Neonatal Lung Injury in Rats. Am J Respir Crit Care Med 180: 1131–1142. 19713449 10.1164/rccm.200902-0179OC PMC3269236

[16] Alphonse RS, Vadivel A, Coltan L, Eaton F, Barr AJ, et al (2011) Activation of Akt protects alveoli from neonatal oxygen-induced lung injury. Am J Respir Cell Mol Biol 44: 146–154. 20348209 10.1165/rcmb.2009-0182OC

[17] Almario B, Wu S, Peng J, Alapati D, Chen S, et al (2012) Pentoxifylline and prevention of hyperoxia-induced lung -injury in neonatal rats. Pediatr Res 71: 583–589. 22322387 10.1038/pr.2012.14

[18] Brown CB, Feiner L, Lu MM, Li J, Ma X, et al (2001) PlexinA2 and semaphorin signaling during cardiac neural crest development. Development 128: 3071–3080. 11688557 10.1242/dev.128.16.3071

[19] Metzger RJ, Klein OD, Martin GR, Krasnow MA (2008) The branching programme of mouse lung development. Nature 453: 745–750. 18463632 10.1038/nature07005 PMC2892995

[20] Roth-Kleiner M, Post M (2005) Similarities and dissimilarities of branching and septation during lung development. Pediatr Pulmonol 40: 113–134. 15965895 10.1002/ppul.20252

[21] Feiner L, Webber AL, Brown CB, Lu MM, Jia L, et al (2001) Targeted disruption of semaphorin 3C leads to persistent truncus arteriosus and aortic arch interruption. Development 128: 3061–3070. 11688556 10.1242/dev.128.16.3061

[22] Becker PM, Tran TS, Delannoy MJ, He C, Shannon JM, et al (2011) Semaphorin 3A contributes to distal pulmonary epithelial cell differentiation and lung morphogenesis. PLoS One 6: e27449. 22096573 10.1371/journal.pone.0027449 PMC3214054

[23] Joza S, Wang J, Fox E, Hillman V, Ackerley C, et al.. (2012) Loss of Semaphorin-Neuropilin-1 Signaling Causes Dysmorphic Vascularization Reminiscent of Alveolar Capillary Dysplasia. Am J Pathol. 10.1016/j.ajpath.2012.08.037 23063659

[24] Le A, Zielinski R, He C, Crow MT, Biswal S, et al (2009) Pulmonary epithelial neuropilin-1 deletion enhances development of cigarette smoke-induced emphysema. Am J Respir Crit Care Med 180: 396–406. 19520907 10.1164/rccm.200809-1483OC PMC2742758

[25] Pasterkamp RJ, De Winter F, Holtmaat AJ, Verhaagen J (1998) Evidence for a role of the chemorepellent semaphorin III and its receptor neuropilin-1 in the regeneration of primary olfactory axons. J Neurosci 18: 9962–9976. 9822752 10.1523/JNEUROSCI.18-23-09962.1998 PMC6793295

[26] Pasterkamp RJ, Giger RJ, Ruitenberg MJ, Holtmaat AJ, De Wit J, et al (1999) Expression of the gene encoding the chemorepellent semaphorin III is induced in the fibroblast component of neural scar tissue formed following injuries of adult but not neonatal CNS. Mol Cell Neurosci 13: 143–166. 10192772 10.1006/mcne.1999.0738

[27] Piaton G, Aigrot MS, Williams A, Moyon S, Tepavcevic V, et al (2011) Class 3 semaphorins influence oligodendrocyte precursor recruitment and remyelination in adult central nervous system. Brain 134: 1156–1167. 21421691 10.1093/brain/awr022

[28] Morishige N, Ko JA, Morita Y, Nishida T (2010) Expression of semaphorin 3A in the rat corneal epithelium during wound healing. Biochem Biophys Res Commun 395: 451–457. 20331965 10.1016/j.bbrc.2010.03.124

[29] Banu N, Teichman J, Dunlap-Brown M, Villegas G, Tufro A (2006) Semaphorin 3C regulates endothelial cell function by increasing integrin activity. FASEB J 20: 2150–2152. 16940438 10.1096/fj.05-5698fje

[30] Vozzelli MA, Mason SN, Whorton MH, Auten RL Jr (2004) Antimacrophage chemokine treatment prevents neutrophil and macrophage influx in hyperoxia-exposed newborn rat lung. Am J Physiol Lung Cell Mol Physiol 286: L488–493. 12588706 10.1152/ajplung.00414.2002

[31] Yi M, Jankov RP, Belcastro R, Humes D, Copland I, et al (2004) Opposing effects of 60% oxygen and neutrophil influx on alveologenesis in the neonatal rat. Am J Respir Crit Care Med 170: 1188–1196. 15347560 10.1164/rccm.200402-215OC

[32] Suzuki K, Kumanogoh A, Kikutani H (2008) Semaphorins and their receptors in immune cell interactions. Nat Immunol 9: 17–23. 18087252 10.1038/ni1553

[33] Husain AN, Siddiqui NH, Stocker JT (1998) Pathology of arrested acinar development in postsurfactant bronchopulmonary dysplasia. Hum Pathol 29: 710–717. 9670828 10.1016/s0046-8177(98)90280-5

[34] Scholzen T, Gerdes J (2000) The Ki-67 protein: from the known and the unknown. J Cell Physiol 182: 311–322. 10653597 10.1002/(SICI)1097-4652(200003)182:3<311::AID-JCP1>3.0.CO;2-9

